# TIMELESS inhibits breast cancer cell invasion and metastasis by down-regulating the expression of MMP9

**DOI:** 10.1186/s12935-021-01752-y

**Published:** 2021-01-11

**Authors:** Bowen Li, Liying Mu, Yanan Li, Kangkai Xia, Yuxi Yang, Sattout Aman, Bashir Ahmad, Shujing Li, Huijian Wu

**Affiliations:** School of Bioengineering & Key Laboratory of Protein Modification and Disease, Liaoning Province, Dalian University of Technology, 2 Ling Gong Road, Dalian, 116024 Liaoning China

**Keywords:** TIMELESS, MMP9, Invasion, Metastasis, Breast cancer

## Abstract

Breast cancer is the first killer leading to female death, and tumor metastasis is one of the important factors leading to the death of patients, but the specific mechanism of breast cancer metastasis is not very clear at present. Our study showed that overexpression of TIMELESS could significantly inhibit the invasion and metastasis of breast cancer cells ZR-75-30 and the assembly of F-actin protein. On the contrary, knockdown of TIMELESS promoted the invasion and metastasis of breast cancer cells. Further study revealed that TIMELESS overexpression decreased the mRNA and protein levels of MMP9. Furthermore, TIMELESS could interact with p65, leading to repress the association of p65 and its acetyltransferase CBP and down-regulating the acetylation level of p65, which inhibited the activation of NF-κB signal pathway. In conclusion, our research showed that TIMELESS may repress the invasion and metastasis of breast cancer cells via inhibiting the acetylation of p65, inhibiting the activation of NF-κB, thus down-regulating the expression of MMP9, and then inhibiting the invasion and metastasis of breast cancer cells.

## Background

Breast cancer is one of the most serious threats to women's health worldwide. Its annual average growth rate is about 4% with poor prognosis and the median survival time is only 2–3 years in China, which is a bad situation and closely related to the invasion and metastasis of breast cancer cells [1, 2]. However, the molecular mechanism is not yet clearly demonstrated. Therefore, research on the mechanisms of breast cancer invasion and metastasis will help provide new strategies for clinical diagnosis and treatment.

In the past, cancer research mainly focused on the mutation of cancer cells to gain the advantage of cell proliferation or survival. At present, more and more evidence show that tumor microenvironment, especially extracellular matrix, has become the main factor affecting the progress of cancer. MMPs are expressed in almost all human cancers [3], such as normal fibroblasts, cancer-related fibroblasts and cancer cells themselves, which can be expressed in the adjacent stroma of tumors. Because MMPs can promote angiogenesis, tumor growth and metastasis, tumor immune regulation and affect the microenvironment before tumor metastasis, the expression of MMP family is closely related to tumor invasion, stage and prognosis [4].

MMP9 is one of the most important members of MMPs family and one of the target genes of NF-κB. It is also called gelatinase B. It can degrade collagen IV, the main component of the cell basement membrane, which plays an important role in cancer metastasis [5]. MMP9 can take different effects in the process of cell scatter, such as tumor invasion, tumor-induced angiogenesis, immune regulation of tumor microenvironment and the formation of microenvironment before metastasis to promote tumor cell distant metastasis [6]. The increased expression of MMP9 is also related to the invasion, metastasis and poor prognosis of different types of cancer, such as cervical cancer, colorectal cancer, ovarian cancer and breast cancer [7–10].

The expression of MMP9 is a rate-limiting step of MMP9 expression. There are many transcriptional factors that can bind with MMP9 promoter, including NF-κB, AP1, SP1, etc. [11]. Therefore, the study of the transcriptional regulation of MMP9 will be favorite to revealing its fine mechanism of action in tumor.

TIMELESS was originally identified in Drosophila melanogaster as an integral part of the circadian rhythm [12]. In mammals, TIMELESS still has the function of regulating daily rhythm, but TIMELESS is best characterized in DNA replication and damage repair [13, 14]. In a normal environment, TIMELESS can ensure the normal replication of DNA. This important role is mainly achieved by controlling DNA replication, maintaining the stability of replication fork and genome [15, 16]. Therefore, TIMELESS is considered to be a member of the replication fork protection complex. In recent years, it has been reported that TIMELESS can interact with PARP-1 to promote homologous recombination repair of DNA double-strand. This process neither depends on poly ADP ribosylation nor affects the enzyme activity of PARP-1. PARP-1 mediates the recruitment of TIMELESS into DSB (DNA double-strand breaks) to promote homologous recombination repair [17, 18]

In our study, TIMELESS inhibited the invasion and metastasis of breast cancer cells by down-regulating the mRNA and protein levels of MMP9. Since MMP9 is a target gene of the NF-κB signaling pathway, whether TIMELESS down-regulates MMP9 is examined through the NF-κB signaling pathway. The study of TIMELESS on the NF-κB signal pathway showed that TIMELESS could inhibit the interaction between NF-κB p65 and its acetyltransferase CBP through the interaction with p65, thereby reducing the acetylation of p65, thus impeding the activation of NF-κB signal pathway.

## Materials and methods

### Cell culture and transfection

HEK-293T, COS-7, ZR-75-30, T47D and MCF-7 cells used in this experiment were all generous gifts from the Cell Resource Center of Dalian Medical University and they are cultured as previously described [19]. Cells were grown at 37 °C in a humidified 5% CO_2_ atmosphere and then the cells were transfected with the appropriate plasmids using Lipofectamine 2000.

### Plasmid construction and antibodies

Human TIMELESS was cloned from a pCMV-Timeless plasmid. The sense and antisense primers were TIMELESS-F: 5′-CCAAGCTTGGGATGGACTTGCACATGATGAAC-3′ and TIMELESS-R: 5′-GCTCTAGAGCTCAGTCATCCTCATCATCCTC-3′, respectively. The prepared TIMELESS DNA fragment was then inserted into the expression vector 3 × FLAG-pcDNA3.1 at the Xba I-Hind III sites. pGL3-MMP9-luc was constructed in my lab before and they both were acquired as previously described [20]. Mouse anti-Flag antibody was purchased from Sigma (M2, 1:8000). Rabbit anti-Timeless was purchased from Abcam (ab72458, 1:6000). Rabbit anti-MMP9 antibodies were purchased from Wanlei (WL02141, 1:500). Rabbit p65 antibody was obtained from Abcam (ab16502, 1:5000). Rabbit GFP antibody was obtained from GeneTex (GTX113617, 1:8000). Mouse anti β-Tublin was purchased from ORIGENE (TA347065, 1:6000). Mouse anti GAPDH was purchased from ORIGENE (TA802519, 1:6000). Rabbit acetylated lysine antibody was obtained from CST (9441, 1:3000).

### Transwell and scratch wound-healing assays

Transwell and scratch wound-healing were performed to access the properties of invasion and metastasis of breast cancer cells and they were operated as previously described [21]. The inoculation amount of the cells in one chamber in the Transwell migration experiment was 2 × 10^4^ and that was 5 × 10^4^ in the Transwell invasion experiment.

### Luciferase reporter assay

Promoter activity was examined by a luciferase assay system. Cells were inoculated in 24-well plates, and cultured for 24 h. Next, the cells were transfected with corresponding plasmids using Lipofectamine 2000 according to the company's specification. Twenty-four hours following the transfection, the cells were subjected to luciferase and Renilla activity assays according to the manufacturer's instructions (Promega, Madison, WI, USA).

### Construction of the shTIMELESS#1 and shTIMELESS#2

TIMELESS is expressed at various levels in different breast cancer tissues. Thus, carrying out a knockdown experiment with a plasmid expression vector of RNA interference targeting TIMELESS is necessary. The pRNAT-U6.1 vector was purchased from GenScript (GenScript, Piscataway, NJ, USA). The sequences of RNA interference were as follows: 5′-GGTTCGAGAGATGACTGAGGGCTAT-3′ (shTIMELESS#1) and 5′-TCCAGGGTAGCTTAGTCCTTTCAAA-3′ (shTIMELESS#2) [24].

### Realtime PCR

The extraction of total RNA and real-time PCR were performed as previously described [22]. The primers for TNF-α mRNA were CCCAGGCAGTCAGATCATCTTC (forward) and AGCTGCCCCTCAGCTTGA (reverse); VEGFA mRNA were CGGGAACCAGATCTCTCACC (forward) and AAAATGGCGAATCCAATTCC (reverse); uPA mRNA were AGTGTCAGCAGCCCCACT (forward) and CCCCCTGAGTCTCCCTGG (reverse); MMP9 mRNA were TACTGTGCCTTTGAGTCCG (forward) and TTGTCGGCGATAAGGAAG (reverse); TIMELESS mRNA were CTCCTCCGGGCTTCTGA (forward) and CCATACATCAGTGGACCAACC (reverse); and glyceraldehyde 3-phosphate dehydrogenase (GAPDH as a reference gene) mRNA were GGGTGTGAACCATGAGAAGT (forward) and GACTGTGGTCATGAGTCCT (reverse). The mRNA level was normalized to GAPDH as the endogenous control. Each target was measured in triplicate.

### The mammalian two-hybrid system

The checkmateTM mammalian two-hybrid system was purchased from Promega. p65 and TIMELESS were subcloned into pBIND at EcoRV/BamHI sites and pACT at Mlu site, respectively.

### Immunoprecipitation and Western blot assay

Western blot assays were conducted as previously described [23].

### Immunofluorescence assay

Immunofluorescence assays were conducted as previously described [23].

### Statistical analysis

Data was examined as means ± SDs. An unpaired t-test was used when the results from the two groups were compared. Statistical analysis was carried out by one-way analysis of variance with Bonferroni's multiple-comparison correction for comparison among three or more groups. Statistical significance was considered at the P < 0.05 level.

## Results

### TIMELESS down-regulates the migration and invasion of breast cancer cells

**Fig. 1:**
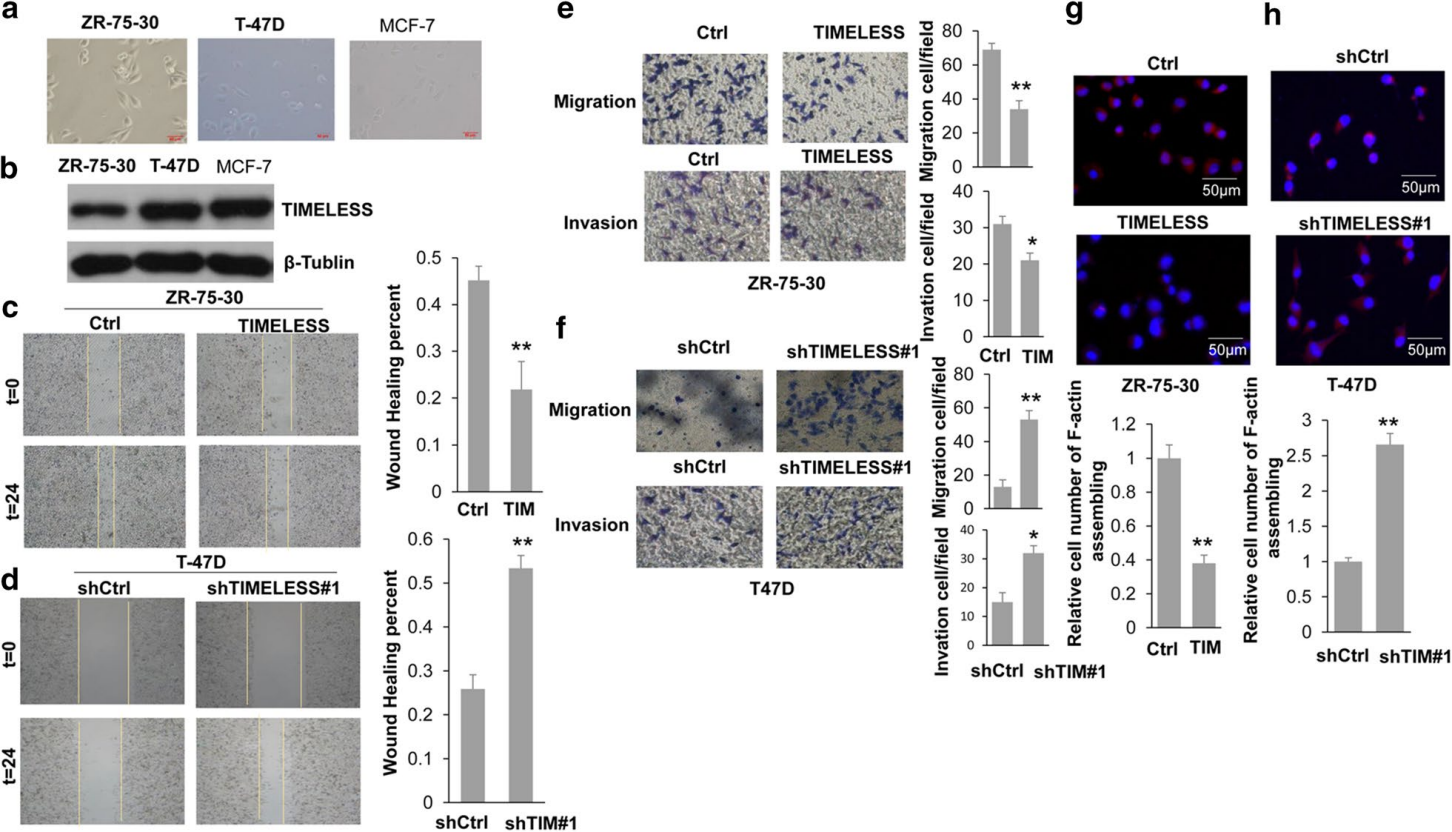
The effect of TIMELESS on the cellular motility of breast cancer cells. **a** Phase-contrast microscopic images of the human breast cancer cell lines. MCF-7, T47D and ZR-75*30. **b** Western blot analysis of TIMELESS expression in the breast cancer cells. **c**, **d** Scratch wound-healing assay assessing the effects of TIMELESS on the motility of breast cancer cells. **e**, **f** Transwell migration and invasion assays evaluating the effects of TIMELESS overexpression (upper) or knockdown (lower) on the cellular motility and invasion ability of breast cancer cells. Cell migration and invasion assays were performed in 24-well chambers without or with Matrigel. Cells were transfected with FLAG-TIMELESS and control vector (Ctrl) or shTIMELESS#1 and control vector (shCtrl) and then plated in the upper chamber. After 24 h of incubation, the migrating and invading cells on the lower surface of the filter were stained and counted. **g**, **h** Immunofluorescence assays accessing the effects of TIMELESS overexpression (left) or knockdown (right) on the F-actin organization of breast cancer cells. 2 × 10^5^ cells were cultured on the cover glass in each new 35 mm cell culture plate. F-actin immunofluorescence was examined and photographed using a Nikon TE2000-U microscope after 24 h-transfection with corresponding plasmids. F-actin was visualized with tetraethyl rhodamine isothiocyanate (TRITC). Nuclei were stained with 4,6-diamidino-2-phenylindole (DAPI). Each bar represents the mean ± s.d. from three independent experiments. “*”, p < 0.05; “**”, p < 0.01

Based on the results of KM analysis on patients of basal-like breast cancer, the survival of patients with high expression of *TIMELESS* was significantly higher than that of patients with low expression (P < 0.05), suggesting that the expression of *TIMELESS* was positively correlated with the survival of patients with basal-like breast cancer (Additional file 1: Figure S1). Basal-like morphology was more often associated with metastatic breast cancer [24]. Three different breast cancer cell morphology was examined and the result showed that in the cell lines with high TIMELESS expression, the cells were mainly epithelioid morphology (Fig. 1a, b), while in ZR-75-30 cell line, the expression of TIMELESS was lower than the other two, and the cell morphology was mesenchymal-like (Fig. 1a, b). This suggested that TIMELESS might play an important role in the invasion and metastasis of breast cancer cells. In ZR-75-30 cells, TIMELESS overexpression could significantly inhibit the speed of scratch healing (Fig. 1c). On the contrary, TIMELESS knockdown accelerated this process in T47D cells (Fig. 1d). Similar results were obtained from the following invasion and metastasis experiments. The overexpression of TIMELESS repressed the invasion and metastasis of breast cancer cells (Fig. 1e), while down-regulating TIMELESS facilitated the invasion and metastasis of breast cancer cells (Fig. 1f). The assembly of intracellular actin into F-actin, a process having an important impact on cell characteristics of motility and invasiveness. Specifically, the cell polarity is reduced or lost and the relatively rounded cells change into a long and narrow shape with F-actin organized along the direction of cell movement, thereby facilitating cell migration from the fixed surface to the free surface [25]. Immunofluorescence experiments showed that the organization of actin was disrupted and not easily integrated into bundles of F-actin with TIMELESS overexpression compared to that of the control group (Fig. 1g). While silencing TIMELESS, F-actin was organized in abundant stressed fibers (Fig. 1h), i.e., overexpression of TIMELESS in breast cancer cells inhibited the organization of loose actin into F-actin while TIMELESS knocking down promoted the assembly process of actin. The role of TIMELESS on the motility of breast cancer cells was not owing to impairing cell proliferation, because the overexpression or knockdown of TIMELESS had no significant effect on cell proliferation within 24 h (Additional file 2: Figure S2 and Additional file 3: Figure S3). The knockdown efficiency of TIMELESS was shown in Additional file 3: Figure S3. To sum up, overexpression of TIMELESS inhibited the invasion and metastasis of breast cancer cells while knocking down TIMELESS promoted these two abilities.

### TIMELESS inhibited the expression of MMP9

**Fig. 2:**
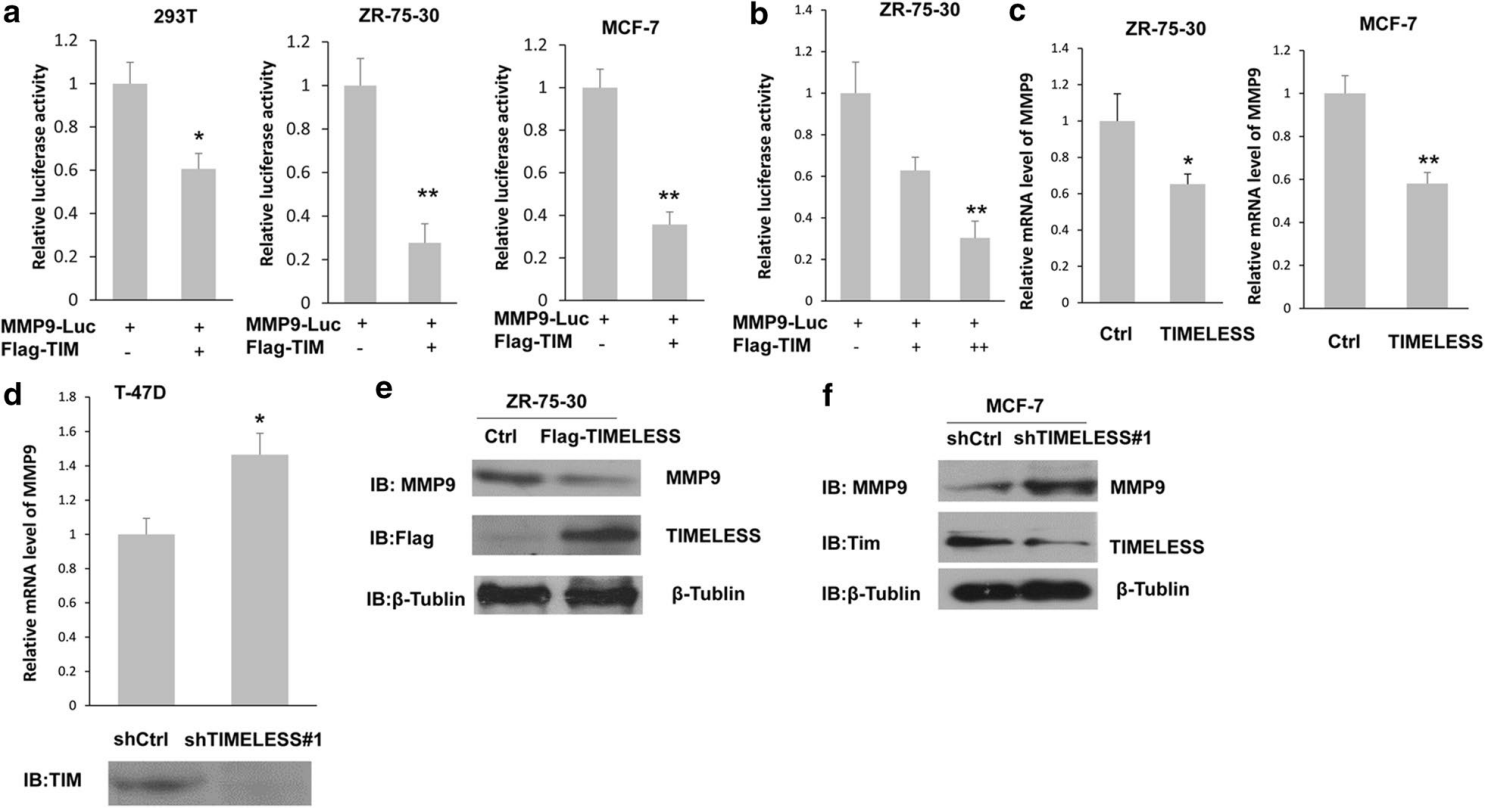
The effect of TIMELESS on the expression of MMP9. **a** The cells were transfected with MMP9 luc with or without Flag-TIMELESS, and then cells were collected and luciferase activity was measured after 24 h transfection. **b** Similar experiment as A was performed but with an increasing dose of Flag-TIMELESS. **c** The breast cancer cells were transfected with a control vector or Flag-TIMELESS for 24 h and then subjected to the real-time PCR analysis. **d** T47D cells were transfected with shcontrol vector or shTIMELESS#1 for 24 h, and then the mRNA level was measured by real-time PCR. **e** ZR-75–30 cells were transfected with control vector or Flag-TIMELESS for 24 h, followed by western blot with anti-MMP9, anti-Flag or anti-β-Tubulin antibodies. **f** MCF-7 cells were transfected with shcontrol vector or shTIMELESS#1 for 24 h, followed by western blot with anti-MMP9, anti-TIMELESS or anti-β-Tubulin antibodies. Each bar represents the mean ± s.d. from three independent experiments. “*”, p < 0.05; “**”, p < 0.01

MMP9 is a key factor in the process of tumor invasion and metastasis. Next, we tested whether TIMELESS could inhibit the invasion and metastasis of breast cancer via The MMP9 signaling pathway. Through the luciferase reporter gene experiment, overexpression of TIMELESS in different cell lines could reduce the transcriptional activity of MMP9 (Fig. 2a), and this inhibition is dose-dependent (Fig. 2b). The mRNA level of MMP9 was also detected and the result showed that the overexpression of TIMELESS decreased the mRNA level of MMP9 (Fig. 2c), on the contrary, TIMELESS knockdown increased the expression of MMP9 (Fig. 2d). Similar results were obtained in the examination of MMP9 protein level in breast cancer cells, that is, the overexpression of TIMELESS increased the expression of endogenous MMP9 (Fig. 2e), while TIMELESS knockdown inhibited the protein level of MMP9 (Fig. 2f). It can be concluded that TIMELESS may negatively regulate the mRNA and protein levels of MMP9.

### TIMELESS repressed MMP9 expression by reducing the activation of NF-κB

**Fig. 3:**
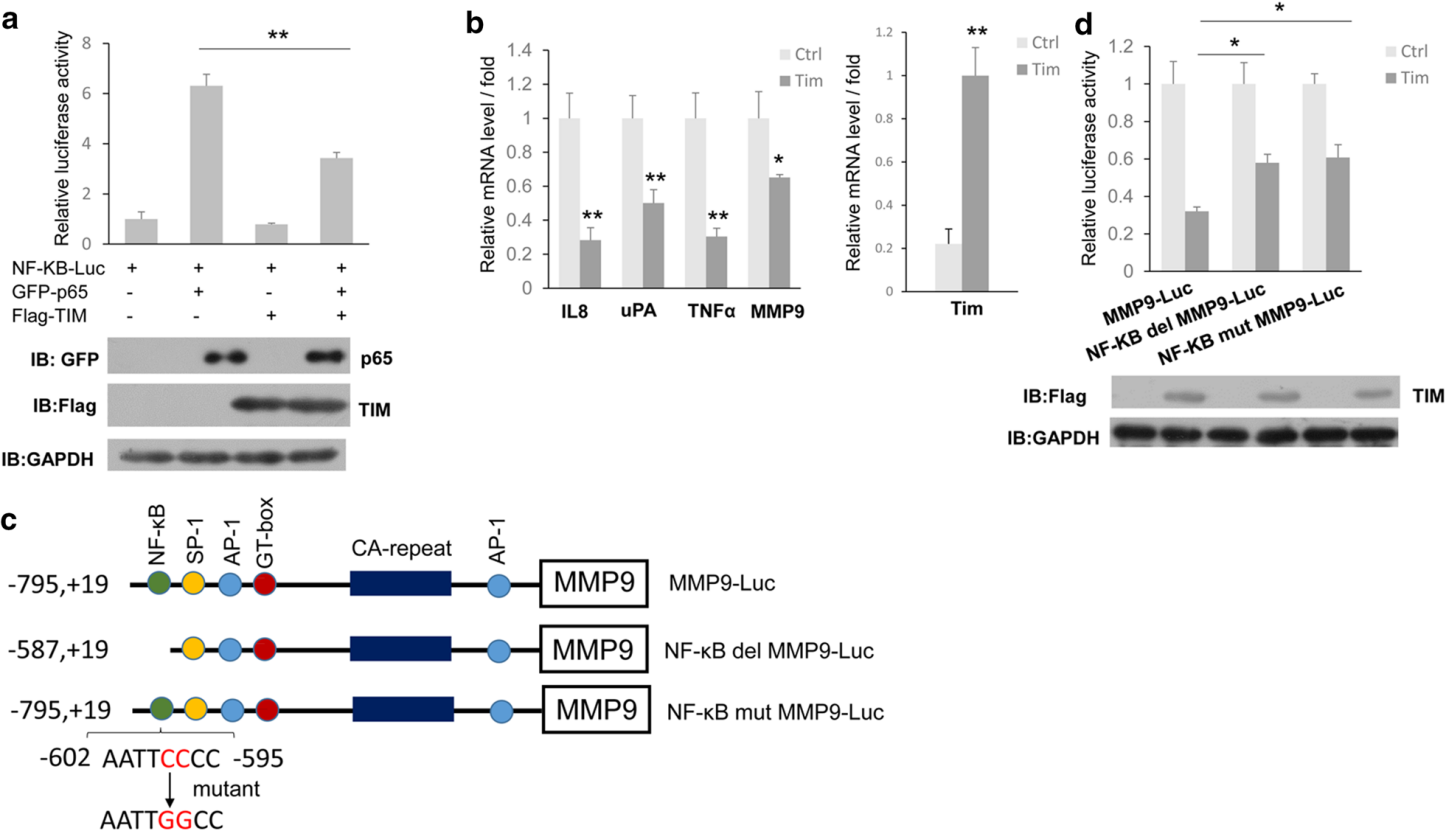
The role of TIMELESS on the activation of NF-κB signal pathway. **a** ZR-75-30 cells were transfected with NF-κB luc and either with p65 or TIMELESS, p65 and TIMELESS for 24 h and then the luciferase activity was measured (up). The cell lysate was subjected to western blot with anti-GFP or anti-Flag antibody (down). **b** ZR-75-30 cells were transfected with control vector or TIMELESS and then subjected to the real-time PCR analysis after 24 h transfection. **c** The schematic of MMP9-Luc, NF-κB del MMP9-Luc, and NF-κB mut MMP9-Luc. **d** ZR-75-30 cells were transfected with MMP9-luc, NF-κB del MMP9-Luc or NF-κB mut MMP9-Luc together with overexpression of TIMELESS or not for 24 h. After that the luciferase activity was measured (up). The cell lysate was subjected to western blot with anti-Flag antibody (down). Each bar represents the mean ± s.d. from three independent experiments. “*”, p < 0.05; “**”, p < 0.01

As we all know, MMP9 is an important downstream target gene of the NF-κB signaling pathway, so we studied whether TIMELESS regulates MMP9 through the NF-κB signaling pathway. First, the effect of TIMELESS on the activation of the NF-κB signaling pathway was examined. Results as shown in Fig. 3a, p65 overexpression alone could activate the NF-κB signal pathway, while overexpression of both p65 and TIMELESS significantly reduced NF-κB reporter gene activity compared with p65 overexpression alone (Fig. 3a). By analyzing the mRNA levels of IL8, uPA and TNF α which are the target genes of NF-κB signal pathway, TIMELESS overexpression downregulated the mRNA levels of NF-κB downstream target genes (Fig. 3b). To determine whether TIMELESS regulated the expression of MMP9 through NF-κB, we constructed another two MMP9 luciferase reporter gene, NF-κB-del-MMP9-luci (deletion of NF-κB binding site in the promoter of MMP9) and NF-κB-mut-MMP9-luci (mutant of NF-κB binding site in the promoter of MMP9). The structural schematic was shown in Fig. 3c. The reporter gene assay showed that TIMELESS decreased 70% activity of the wild-type MMP9 reporter gene, while TIMELESS only decreased 40% activity of the two mutants (Fig. 3d), indicating that TIMELESS may inhibit the expression of MMP9 through NF-κB binding site.

### TIMELESS interacted with NF-κB p65

**Fig. 4:**
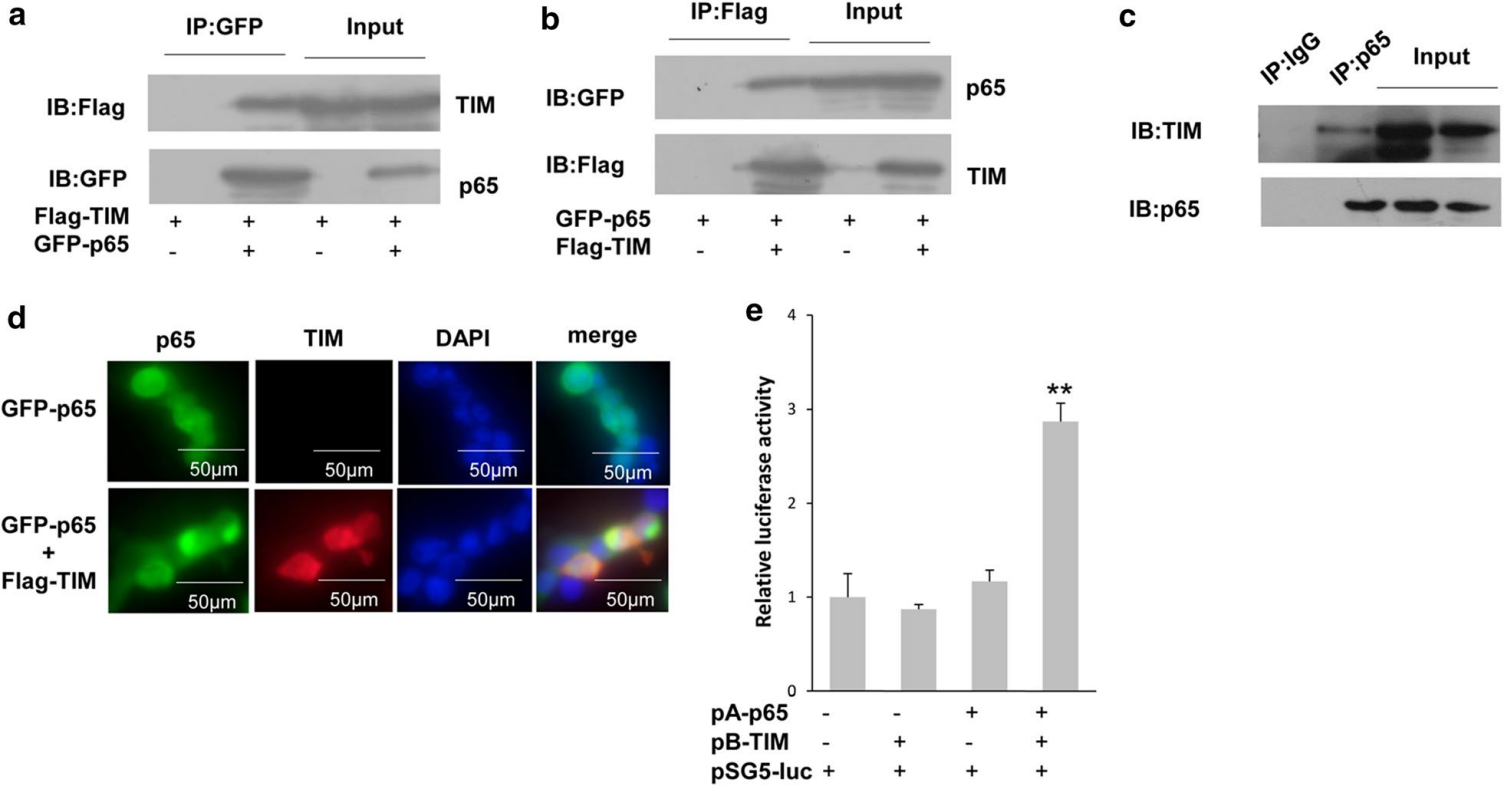
The interaction between p65 and TIMELESS. **a** 293T cells were transfected with Flag-TIMELESS alone with or without GFP-p65 for 24 h, and then subjected to the immunoprecipitation with anti-GFP-antibody followed by western blot with anti-Flag antibody. **b** 293T cells were transfected with the indicated vectors for 24 h and then the immunoprecipitation was performed. **c** Extract from T47D cells was immunoprecipitated with anti-IgG or anti-p65 antibodies followed western blot with anti-TIMELESS antibody. **d** T47D cells were transfected with GFP-p65 together with or without Flag-TIMELESS for 24 h. TIMELESS was stained with anti-Flag antibody and tetramethylrhodamine isothiocyanate (TRITC)-conjugated anti-rabbit IgG, and p65 was visualized with green fluorescence. Nuclei were stained with 4,6-diamidino-2-phenylindole (DAPI). **e** The direct interaction between p65 and TIMELESS was identified through mammalian two-hybrid systems. ZR-75–30 cells were transfected with the indicated vectors for 36 h and the activity of pSG5 luciferase was measured. Each bar represents the mean ± s.d. from three independent experiments. “*”, p < 0.05; “**”, p < 0.01

Since TIMELESS could inhibit the activation of NF-κB signaling pathway, we examined whether TIMELESS could interact with p65, which is a member of NF-κB. The immunoprecipitation experiment identified exogenous TIMELESS could interact with p65 (Fig. 4a, b). Immunoprecipitated p65 from T47D cells was detected by anti-TIMELESS antibody, indicating the interaction between TIMELESS and p65 (Fig. 4c). Through the immunofluorescence experiment, TIMELESS and p65 were mainly distributed in the nucleus. At the same time, the overexpression of TIMELESS does not affect the location of p65 in the cell (Fig. 4d). The direct interaction between TIMELESS and p65 was studied via mammalian two-hybrid systems in ZR-75-30 cells. Compared with the control group, the co-expression of TIMELESS and p65 resulted in a twofold increase of luciferase reporter gene (Fig. 4e), indicating that there is direct interaction between the two proteins in cells. This suggested that TIMELESS may participate in the NF-κB signal pathway via binding with p65.

### TIMELESS inhibits the acetylation of p65 by inhibiting its interaction with its acetyltransferase CBP

**Fig. 5:**
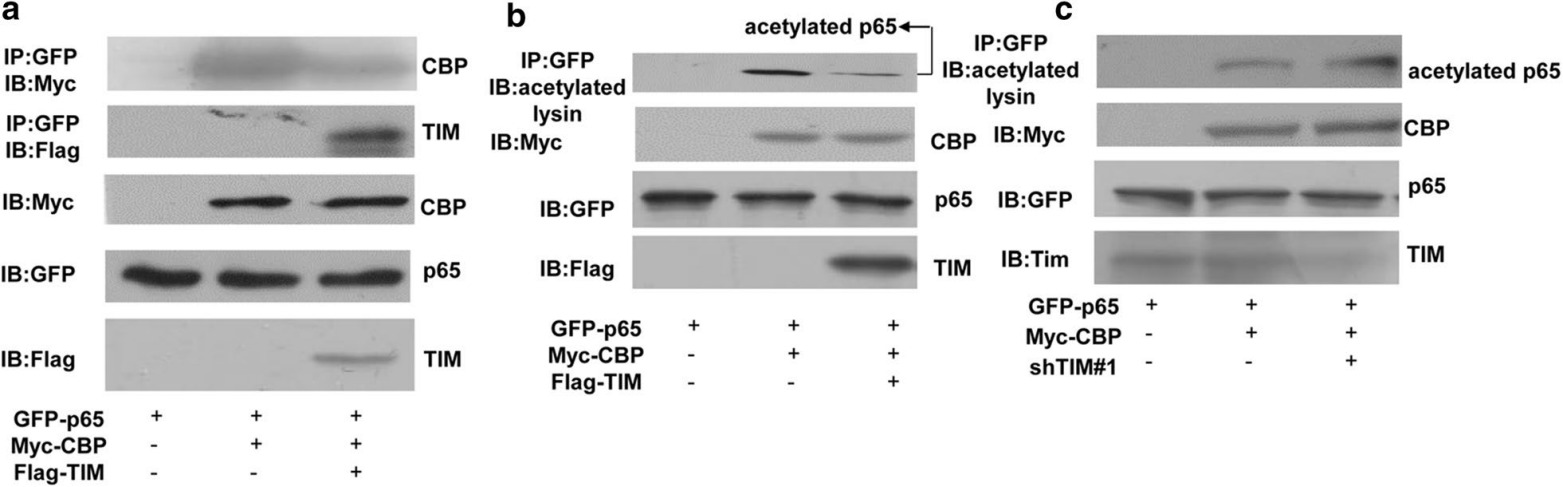
TIMELESS decreased the acetylation of p65. **a** 293T cells were transfected with GFP-p65 only, GFP-p65 and Myc-CBP or GFP-p65, Myc-CBP and Flag-TIMELESS for 24 h. The extract was subjected to immunoprecipitation with anti-GFP antibody followed by western blot with anti-Myc or anti-Flag antibodies. **b** 293T cells were transfected with the vectors as described above. Cell lysate was immunoprecipitated with anti-GFP antibody followed by western blot with anti-acetylated lysine antibody. **c** T47D cells were transfected with GFP-p65 only, GFP-p65 and Myc-CBP together with or without shTIMELESS#1 for 24 h. The extract was subjected to immunoprecipitation with anti-GFP antibody followed by western blot with anti- acetylated lysine antibody

The acetylation of p65 is considered to be a crucial condition of activation of NF-κB signaling pathway, and CBP is the classical acetyltransferase of p65 [26]. Therefore, whether the overexpression of TIMELESS affects the interaction between p65 and its acetyltransferase CBP is detected. It can be seen from Fig. 5a that when TIMELESS and CBP are co-expressed, the interaction band of p65 and CBP is obviously weakened compared with the overexpression of CBP alone (Fig. 5a), indicating that TIMELESS inhibited the interaction between p65 and its acetyltransferase. The detection of the acetylation level of p65 showed that the overexpression of TIMELESS did decrease the acetylation level of p65 (Fig. 5b), while the acetylation level of p65 in cells with TIMELESS knockdown was higher than that in the control group (Fig. 5c). These data indicated that TIMELESS can reduce the acetylation of p65 through inhibiting the association of the acetyltransferase CBP and p65, and then repress the activation of NF-κB signal pathway.

## Discussion

Metastasis is the main cause of unsuccessful treatment, recurrence and death of breast cancer patients. Therefore, it is an urgent problem to search for an effective metastasis target of breast cancer. Because of the relatively high expression of MMP9 in patients with high invasive breast cancer, it is of great clinical significance to study the regulation mechanism of MMP9 expression. TIMELESS, first discovered in Drosophila, has been found in recent years to regulate various cellular functions, embryonic development, and initiation and progress of cancer, apart from regulating the circadian rhythm of organisms [27]. TIMELESS plays an important role in the development of breast cancer, mainly in its influence on the proliferation ability of breast cancer cells [28]. However, there are few reports regarding the mechanisms on TIMELESS regulating the invasive and metastatic ability of breast cancer cells. Our study reveals a new molecular regulatory mechanism, that is, TIMELESS can inhibit breast cancer migration and invasion by reducing the transcription activity of MMP9, and systematically expounds the molecular mechanism of TIMELESS regulating NF-κB/MMP9 pathway. It provides a theoretical basis for the clinical use of MMP9 as a target to inhibit metastasis by inducing TIMELESS.

Metastasis is a main biological characteristic of malignant tumors, and also the main cause of death. The occurrence and development of tumor not only depends on tumor cell itself but also depends on the survival environment of tumor cell, namely tumor microenvironment [29]. More and more researches focus on the role of tumor microenvironment in tumorigenesis, development, invasion and metastasis. MMPs are a group of zinc and calcium-dependent endopeptidases, which is composed of more than 20 members. It is an important protease for degradation of extracellular matrix and plays a key role in tumor proliferation, angiogenesis, invasion and metastasis. A large number of reports showed that MMPs were highly expressed in metastatic tumors. Matrix metalloproteinase-9 (MMP-9) is an important member of the MMPs family. It mainly degrades collagen IV, collagen V and gelatin, and plays a crucial role in the metastasis of the tumor. The expression of MMP-9 expression is positively correlated with lymph node metastasis and histological grade of endometrial carcinoma [30], and negatively correlated with invasion, metastasis and bone metastasis of breast cancer [31]. The pathological analysis of MMP-9 in primary ureteral carcinoma showed that the expression of MMP-9 was significantly higher than that in normal tissues [32].

In recent years, researchers have detected that the expression level of TIMELESS is often significantly higher or lower in cancerous tissues than that of non-cancerous tissues adjacent to the tumor in various organs cancer. More studies have proved that it may be related to the progression of cancer. For example, TIMELESS can boost the development of liver cancer [33]. TIMELESS overexpression in lung cancer is associated with low patient survival [34]. TIMELESS can be used as a pathological marker in colorectal cancer [35], etc. For breast cancer, TIMELESS regulates the ribosylation of ERα and activates cell proliferation-related signaling pathways by acting as a novel cofactor for Erα [28]. However, there are few studies on the regulation mechanism of invasion and metastasis in breast cancer cells by TIMELESS. Our work shows that TIMELESS can inhibit the invasion and metastasis of breast cancer cells by decreasing the expression of MMP9 (Figs. 1 and 2). This gives TIMELESS a new role in the field of the tumor and provides a basis for further revealing the fine regulatory mechanism of MMP9.

NF-κB pathway is involved in many pathological processes of carcinogenesis, including proliferation, cell survival, apoptosis, invasion, angiogenesis and inflammation. There are five members in NF-κB family, and p65/P50 complex plays an important role in NF-κB signal pathway. In the classical NF-κB signaling pathway, IKB which is the repressor of p65/p50 is degraded due to ubiquitination with stimulation of activation signal, thus relieving the inhibition of p65/p50 and promoting p65/p50 to play a role in transcriptional activation. Numbers of target genes are regulated by p65/ P50 such as c-myc, p53, TNF-a, COX-2, VEGF, uPA, IL-8 and MMPs, etc. [36]. Post-translational modifications of p65 confer more complexity and functional diversity on the NF-κB pathway. Among these modifications, phosphorylation of serine-threonine residues at p65 specific sites is considered to play an important role in transcriptional activation [37]. In addition, the acetylation of p65 is also closely related to the activation of NF-κB [26]. Our results show that TIMELESS can form complex with p65 and CBP, thus inhibiting the interaction between p65 and its acetyltransferase CBP (Fig. 5a), and then reducing the acetylation level of p65 (Fig. 5b). The detection of NF-κB downstream target gene showed that TIMELESS overexpression did reduce the mRNA level of NF-κB downstream target gene (Fig. 3b). Therefore, TIMELESS may be a new regulatory factor of NF-κB signaling pathway and participate in the activation of NF-κB signaling pathway.

It has been reported that an NF-κB binding site localized in the promoter of MMP9 gene. The mutation or deletion of NF-κB binding site does reduce the inhibition ratio of TIMELESS to MMP9 promoter but does not completely release the inhibition of MMP9 gene transcription. This suggests that there may be some other ways for TIMELESS to down-regulate the expression of MMP-9. TIMELESS contains not only a classical TIMELESS domain but also three DDT domains [27]. The DDT domain has a DNA binding ability, indicating that TIMELESS may play a role as a transcription factor in associating with DNA localized in the promoter of MMP9. But this work needs to be further studied.

Once the tumor metastasizes, the treatment of cancer is easily out of control. Therefore, the search for new molecular targets for precise treatment has been a hot topic. In this study, a novel molecular mechanism of TIMELESS expression regulating the invasion and metastasis of breast cancer is acquired, which may provide a new basis for the treatment of metastatic breast cancer. It is expected that there will be more in-depth research in this area in the future to provide a more solid theoretical foundation for the treatment of malignant tumors.

## Conclusions

In this study, we identified that TIMELESS may repress the invasion and metastasis of breast cancer cells via inhibiting the acetylation of p65, inhibiting the activation of NF-κB, thus down-regulating the expression of MMP9, and then inhibiting the invasion and metastasis of breast cancer cells.

## Supplementary Information

**Additional file 1: Figure S1.** The correlation between the expression of Timeless and the survival of patients with Basal-like breast cancer by KM analysis. The survival analysis of 239 Basal-like breast cancer patients was performed by Kaplan–Meier method, and the overall survival (OS) was the time of death or last follow-up. Comparisons on difference between groups were based on the Log-rank test, and P < 0.05 was identified as a statistically significant difference. The cut-off values involved in the results of data analysis are the optimal values selected for each group of high-low expression, corresponding to the optimal values of the ROC curve (receiver operating characteristic). The optimal value calculation, data analysis and picture drawing are all done in the database through Kaplan Meier-plotter database (USA).

**Additional file 2: Figure S2.** A and B. MTT assay evaluating the effect of TIMELESS on the proliferation of breast cancer cells. Cells were transfected with FLAG-TIMELESS and control vector (Ctrl) or shTIMELESS#1 and control vector (shCtrl). After 24 h, the cells were performed to the MTT assay according to the manufacturer’s instructions.

**Additional file 3: Figure S3.** The knockdown efficiency of shTIMELESS. T47D cells were transfected with shcontrol, shTIMELESS#1 or shTIMELESS#2 for 24 h, and then the cell lysis was subjected to western blot with anti-TIMELESS or GAPDH antibodies.

## Data Availability

Please contact the corresponding author for all data requests.

## Abbreviations

CBP: CREB binding protein
MMP9: Matrix metalloproteinase9
PARP1: Poly (ADP-ribose) polymerase-1
DSB: DNA double-strand breaks
TNF-α: Tumor necrosis factor α
COX-2: Cyclo-oxygen-ase-2
VEGF: Vascular endothelial growth factor
