# Supplementary figures and images for: TIMELESS inhibits breast cancer cell invasion and metastasis by down-regulating the expression of MMP9
Source: Cancer Cell Int. 2021 Jan 11;21:38. doi: 10.1186/s12935-021-01752-y (PMC7798230; doi:10.1186/s12935-021-01752-y)

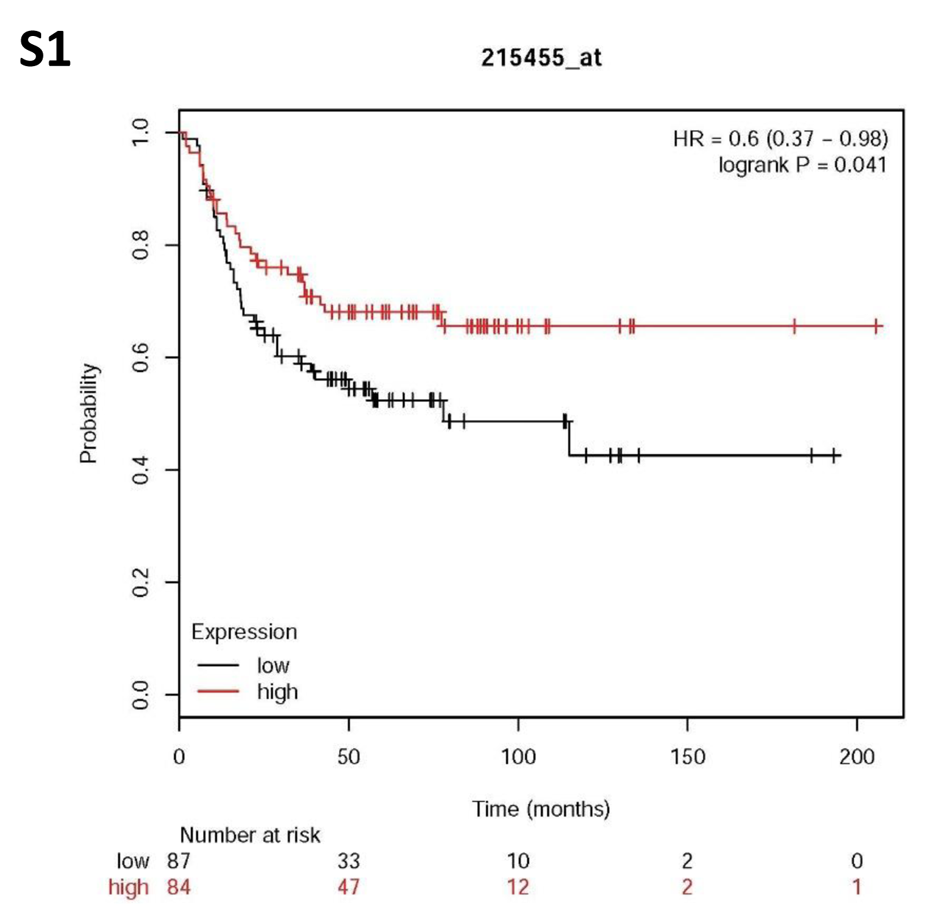

Supplement: Supplementary file 1 — Additional file 1: Figure S1. The correlation between the expression of Timeless and the survival of patients with Basal-like breast cancer by KM analysis. The survival analysis of 239 Basal-like breast cancer patients was performed by Kaplan–Meier method, and the overall survival (OS) was the time of death or last follow-up. Comparisons on difference between groups were based on the Log-rank test, and P < 0.05 was identified as a statistically significant difference. The cut-off values involved in the results of data analysis are the optimal values selected for each group of high-low expression, corresponding to the optimal values of the ROC curve (receiver operating characteristic). The optimal value calculation, data analysis and picture drawing are all done in the database through Kaplan Meier-plotter database (USA). [file 12935_2021_1752_MOESM1_ESM.tif]

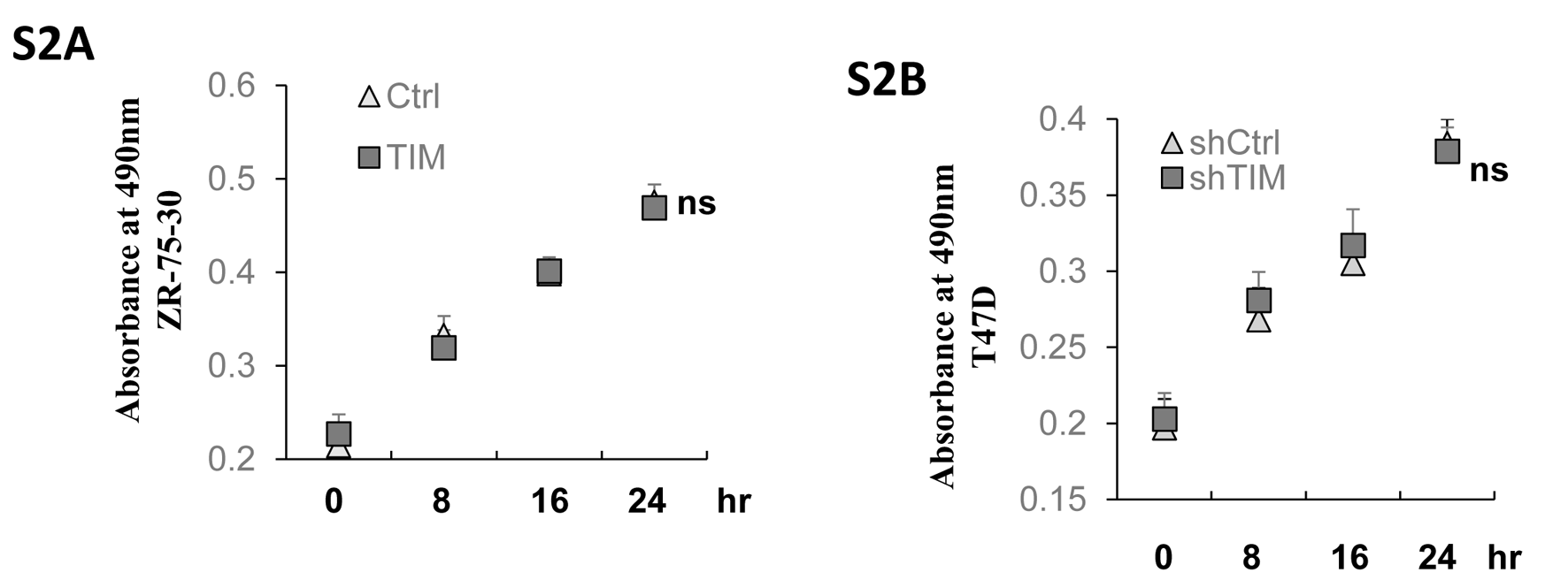

Supplement: Supplementary file 2 — Additional file 2: Figure S2. A and B. MTT assay evaluating the effect of TIMELESS on the proliferation of breast cancer cells. Cells were transfected with FLAG-TIMELESS and control vector (Ctrl) or shTIMELESS#1 and control vector (shCtrl). After 24 h, the cells were performed to the MTT assay according to the manufacturer’s instructions. [file 12935_2021_1752_MOESM2_ESM.tif]

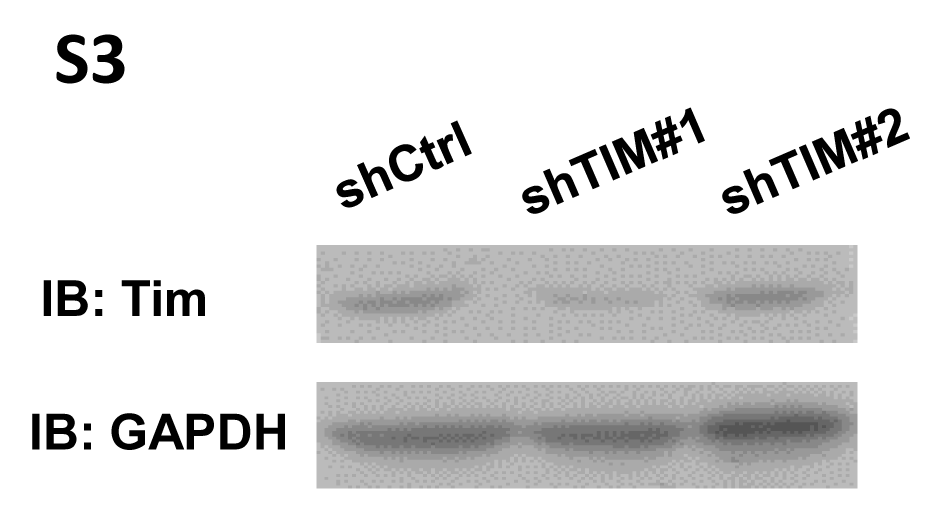

Supplement: Supplementary file 3 — Additional file 3: Figure S3. The knockdown efficiency of shTIMELESS. T47D cells were transfected with shcontrol, shTIMELESS#1 or shTIMELESS#2 for 24 h, and then the cell lysis was subjected to western blot with anti-TIMELESS or GAPDH antibodies. [file 12935_2021_1752_MOESM3_ESM.tif]
